# Interpreting VExUS and renal outcomes: the importance of construct validation in surgical ICU cohorts

**DOI:** 10.1186/s40635-026-00959-5

**Published:** 2026-08-14

**Authors:** Sajid Kadir, Travis Murphy

**Affiliations:** 1 Divisions of Cardiovascular Medicine and Pulmonary and Critical Care Medicine, University of Florida College of Medicine, 655 W 8th Street, Jacksonville, 32209 FL USA; 2 Department of Surgery, University of Miami, Miami, FL USA

## Dear editor

We read with interest the study by Evezard et al. [1] examining the association between the Venous Excess Ultrasound (VExUS) score and worsening renal function (WRF) during loop diuretic therapy in a cardiovascular ICU. The authors report no significant association between VExUS-defined congestion (VExUS > = 2) and WRF, diuretic response, or hemoconcentration. While the clinical question is important and the authors are commendably transparent about their study’s limitations, we believe the null result cannot be confidently interpreted without first establishing that the VExUS classification captured the physiological state it was designed to identify in this particular cohort. We raise three considerations: discordance between the VExUS classification and established congestion markers, the potential influence of unmonitored postoperative atrial fibrillation on Doppler waveform interpretation, and temporal separation between exposure measurement and outcome assessment.

**Discordance between VExUS classification and established congestion markers.** The VExUS score quantifies the downstream organ-level hemodynamic consequences of elevated right atrial pressure (RAP), which is the upstream driver of systemic venous congestion. Elevated RAP transmits backward pressure through the hepatic, portal, and renal venous beds, producing the Doppler flow abnormalities that VExUS captures. This physiological relationship has been validated empirically: Douglas et al. [2] demonstrated that VExUS correlates strongly with invasively measured RAP, with an area under the curve of 0.99 (95% CI 0.96-1.00) for predicting RAP > = 12 mmHg. Similarly, a recent meta-analysis confirms a significant association between elevated VExUS grades and acute kidney injury across multiple observational cohorts [3]. However, in the present study, central venous pressure did not differ between VExUS-defined congestive and non-congestive groups (14 [12–16] vs. 14 [9–16] mmHg, *p* = 0.130), nor did NT-pro-BNP (3540 [1468–6818] vs. 2489 [693–5134] pg/mL, *p* = 0.147). Neither the established invasive marker of venous congestion nor the gold-standard heart failure biomarker validated the VExUS-based group assignment. The authors do not report the methodology of CVP measurement, specifically whether readings were obtained simultaneously with the VExUS assessment, at end-expiration, or with standardized transducer positioning. In mechanically ventilated post-surgical patients, CVP values obtained without standardized methodology are a recognized source of error, and their concordance with the ultrasound assessment cannot be assumed. This discordance does not necessarily invalidate the VExUS score itself, but it does raise the question of whether the binary classification separated patients into physiologically distinct groups in this cohort. If the exposure variable did not reliably discriminate congestion, the null association with WRF is expected regardless of whether a true relationship exists.

**Cardiac rhythm may have compromised VExUS reliability.** The original VExUS grading system was developed and validated in post-cardiac surgery patients in sinus rhythm [4], and the Doppler waveform criteria for hepatic, portal, and renal venous flow assume regular cardiac cycling. The present cohort was 73% post-cardiac surgery, a population in which new-onset postoperative atrial fibrillation occurs in 20–40% of cases, typically within the first 72 h [5], precisely the monitoring window used for VExUS classification. While permanent atrial fibrillation was an exclusion criterion, paroxysmal postoperative atrial fibrillation was not, and cardiac rhythm at the time of each VExUS assessment is not reported. Atrial fibrillation diminishes the hepatic vein S wave and abolishes the A wave, producing an S < D pattern that mimics venous congestion even in the absence of elevated right atrial pressure, a pattern classified as mildly abnormal under the VExUS grading system. Additionally, irregular R-R intervals compromise the reliability of portal and intrarenal venous Doppler measurements, which require averaging over consistent cardiac cycles. An unknown proportion of VExUS scores in this cohort may therefore reflect rhythm-related artifact rather than true venous congestion, potentially misclassifying patients between groups.

**Temporal separation between exposure and outcome.** VExUS was assessed serially at inclusion, 2 h, and 24 h, with patients classified by the maximum score across these timepoints. Notably, two of the three assessments occurred after diuretic therapy had already been initiated, meaning the congestion state captured may already have been modified by the intervention. Moreover, WRF was assessed at ICU discharge (median stay 5 days, IQR 3–10), introducing a substantial temporal gap between the day-1 congestion classification and the renal outcome. During the intervening days, patients received ongoing ICU interventions that collectively determine renal trajectory. This temporal separation makes it difficult to attribute the renal outcome to the congestion state captured by VExUS.

Together, these observations carry implications for future study design. They suggest that the null findings may reflect limitations of VExUS classification fidelity in this specific cohort rather than a true absence of association between venous congestion and renal outcomes. We would encourage future studies examining VExUS-guided diuretic therapy to incorporate: concurrent invasive hemodynamic validation of VExUS group assignment; verification and documentation of cardiac rhythm at each ultrasound assessment; and temporal alignment between congestion measurement and renal outcome, ideally with serial VExUS assessments linked to longitudinal renal biomarkers.

## Data Availability

Not applicable. This correspondence is a commentary on a previously published article and does not report original data. No new datasets were generated or analysed.
